# On the Structure and Growth of Dentine

**Published:** 1877-09

**Authors:** Chr. Sihler


### THE DENTAL REGISTER.

VOL. XXXI.] SEPTEMBER, 1877. [NO. 9.

### ON THE STRUCTURE AND GROWTH OF DENTINE.

BY CHR. SIHLER, M. D.

To study living and growing dentine, human teeth recently extracted, or the incisor teeth of a calf are very convenient, also the permanent incisor which may be obtained from sheep four to six months or about a year of age, have some advantages for investigation; the latter may be injected from the artery entering the inferior dental canal. The calf's teeth after their removal from the jaw, are to be kept in Beale's carmine stain for from ten to fourteen days, a little ammonia having been added to the stain and a portion of the root having been split off with a scalpel, so that the pulp may be enabled to imbibe the coloring matter which it is very slow to do, as are also the dentinal canals or more accurately their contents, but at times one will succeed to stain these also. After the staining is sufficient the teeth are washed off, and then kept in dilute glycerine, acidulated with a little acetic acid for a week. Then they are thrown into dilute alcohol, and finally pure alcohol, so that the pulp may shrink gradually, and when it finally has dried up completely it will adhere in some places to the dentine, so that sections can then be made through dentine and pulp with every part pretty well in its natural relations. Care must be taken that the pulp is not detached from the dentine all around, and to prevent this it is well to loosen the pulp from some portion, and split the root longitudenally that the continuity between the pulp and the dentine may be preserved at some points at least.

Sections may now be made with a hard scalpel through the dentine of the root with the adherent soft parts (pericementum and pulp) in place: the dentine, in the calf's teeth having not yet attained its fullest hardness, and being also softened by the glycerine and acetic acid. The sections are then placed in acetic acid and glycerine, and allowed to swell out. Through the developing permanent incisors of a sheep, sections may be made after the limesalts have been removed by very dilute muriatic acid.

The following observations were made, and can be made upon investigating the above named material:

1. It will be noticed that in the calf's tooth the pulp is very liable to be detached from the dentine, and that there is a great difference in this respect between the pulp and dentine on the one hand, and the cementum and pericementum on the other. While it is difficult to preserve the connection between the dentine and pulp, it is impossible to separate the pericementum from the cementum except by scraping it off.

2. The pulp is very richly supplied with blood vessels, as can well be seen with the naked eye, or after injecting.

3. The pulp although quite succulent, is by no means a very delicate tissue, but is quite resistent to the touch. It will readily tear up lengthwise when picked with needles, and under the microscope appears to be made up of fine fibres to which bioplasts are attached, the fibres appearing imbedded in a softer material.

4. When sections are made longitudinally through the dentine and pulp attached, there can be seen, first, the dentine as described above; second, a layer of dentine often stained pink, which is ready to be infiltrated with limesalts adjoining this; third, comes in thicker sections a dark red zone; and fourth, along side of the dark red zone, the general mass of the pulp, appearing indistinctly fibrous, the fibres always running in a longitudinal direction, and the bioplasts imbedded in this fibrous tissue being stretched more or less in the same direction.

5. In cross sections there will also appear the calcified and uncalcified dentine, the dark red zone lining the latter and the pulp, but the fibrous appearance is of course not observed in the latter.

6. When the dark red zone is more closely examined in these sections, it can be seen to consist of elements (cr bodies.) which have a more or less oblong form, and in stained specimens they present as the base or extremity towards the pulp cavity a dark red spot (or nucleus) on the base on the extremity towards the pulp cavity, while the general mass of the element is of a light red appearance. This is of course the odontoblast of the books.

7. These odontoblasts I have found—upon making sections through dentine with these in place—of very various length.

8. These odontoblasts may be obtained by themselves, by scraping the surface of the recently formed dentine. At times, namely, when the pulp is removed from the dentine, the odontcblasts will remain attached to the dentine, at times they will be removed with the pulp.

9. When so detached, the bioplasts may be examined under high powers, and I have always found, when they were thus separated, and each one by itself, that the dark red mass was at one extremity, forming, so to say, the base. Never have I seen it in any other place. It appears to me either as a flat disk just covering the odontoblasts, or it appears as though the odontoblasts were open at the base, and the dark red contents appeared naked there, while along the sides of the body of the bioplast the light red color might be explained by a membrane covering the deeper portions. Pressure, however, makes the former view more probable. The odontoblasts appeared wider or thicker at the extremity towards the pulp, and tapered off toward the dentinal end. Fine glistening fibres were frequently observed. In some groups the fibres being attached frequently to one angle of the dentinal extremity, at times fine fibres are also seen attached to the side of the odontoblast.

10. Not unfrequently I have succeeded in staining the contents of the dentinal tubes.

11. When sections are made through the pulp and dentine, where there has no separation occured between them, and the preparation being placed in acetic or dilute muriatic acid, the pulp is slowly, through pressure upon the covering glass, separated and pushed off from the dentine, threads apparantly can often be seen running from the pulp into the dentine.

12. In such preparations it can often be seen that where the separation is but slight, the fibres running from the pulp into the dentine, appear much broader than where the distance between the pulp and dentine is greater. Where the distance is greater, they appear often as exceedingly fine threads.

13. When pulp, for example, in longitudinal sections through the sheep's permanent incisor, is detached altogether from the dentine, processes are seen standing out from it, having a granular appearance and of the size of dentinal canals.

14. Tomes says: "At present it admits of doubt, whether they are solid or tubular. When accidentally stretched between two fragments of dentine, the diameter of the fibril becomes much diminished, and when broken across a minute globule of transparent but dense fluid may sometimes be seen at the broken end gathered into a more or less spherical form. These appearances may be explained by assuming that the fibril consists of a sheath containing a semi-fluid matter.

15. Where sections of dentine with the odontoblasts attached are made, and treated with strong muriatic acid, it can be seen that the fibres and the odontoblasts are in continuity. It can also be observed that the odontoblasts are united laterally, and cannot well be separated one from another.

16. Lent has succeeded in isolating single odontoblasts in direct continuity with the dentinal tubule. I have succeeded in this also.

17. When a section through the end of a root of a calf's incisor is made, there will be found in the outside of the finished cementum, a large mass of uncalcified cementum, which aside from the bioplasts imbedded in it, looks quite homogeneous. But when such a section is treated with strong muriatic acid, there will appear a network of apparently fibre, connecting the bioplasts, corresponding to the canaliculi which would become apparent as soon as the homogeneous glue would have been infiltrated with limesalts.

